# Activation of α7 nicotinic acetylcholine receptor by nicotine selectively up-regulates cyclooxygenase-2 and prostaglandin E_2 _in rat microglial cultures

**DOI:** 10.1186/1742-2094-2-4

**Published:** 2005-01-25

**Authors:** Roberta De Simone, Maria Antonietta Ajmone-Cat, Daniela Carnevale, Luisa Minghetti

**Affiliations:** 1 Department of Cell Biology and Neurosciences, Section of Degenerative and Inflammatory Neurological Diseases, Istituto Superiore di Sanità, Rome, Italy

**Keywords:** Brain macrophages, inflammation, TNF, IL-10, Prostaglandin E_2_

## Abstract

**Background:**

Nicotinic acetylcholine (Ach) receptors are ligand-gated pentameric ion channels whose main function is to transmit signals for the neurotransmitter Ach in peripheral and central nervous system. However, the α7 nicotinic receptor has been recently found in several non-neuronal cells and described as an important regulator of cellular function. Nicotine and ACh have been recently reported to inhibit tumor necrosis factor-α (TNF-α) production in human macrophages as well as in mouse microglial cultures. In the present study, we investigated whether the stimulation of α7 nicotinic receptor by the specific agonist nicotine could affect the functional state of activated microglia by promoting and/or inhibiting the release of other important pro-inflammatory and lipid mediator such as prostaglandin E_2_.

**Methods:**

Expression of α7 nicotinic receptor in rat microglial cell was examined by RT-PCR, immunofluorescence staining and Western blot. The functional effects of α7 receptor activation were analyzed in resting or lipopolysaccharide (LPS) stimulated microglial cells pre-treated with nicotine. Culture media were assayed for the levels of tumor necrosis factor, interleukin-1β, nitric oxide, interleukin-10 and prostaglandin E_2_. Total RNA was assayed by RT-PCR for the expression of COX-2 mRNA.

**Results:**

Rat microglial cells express α7 nicotinic receptor, and its activation by nicotine dose-dependently reduces the LPS-induced release of TNF-α, but has little or no effect on nitric oxide, interleukin-10 and interleukin-1β. By contrast, nicotine enhances the expression of cyclooxygenase-2 and the synthesis of one of its major products, prostaglandin E_2_.

**Conclusions:**

Since prostaglandin E_2 _modulates several macrophage and lymphocyte functions, which are instrumental for inflammatory resolution, our study further supports the existence of a brain cholinergic anti-inflammatory pathway mediated by α7 nicotinic receptor that could be potentially exploited for novel treatments of several neuropathologies in which local inflammation, sustained by activated microglia, plays a crucial role.

## Background

The inflammatory response is in the first instance a mechanism of self-defense, set by the innate immune system against endogenous and exogenous insults, and essential for the survival of the organism. Inflammation must be tightly regulated as deficiency as well as excess in its response will result in pathological conditions, such as immunodeficiency or chronic inflammatory diseases [1]. In the last decade increasing evidence has highlighted the role of inflammation in most brain pathologies, including immune-mediated diseases such as multiple sclerosis, acute neurodegeneration following ischemia or trauma, and, more recently, chronic neurodegenerative diseases [2].

Among the endogenous mechanisms that regulate the inflammatory response, cross-talk between the immune and nervous systems play an important role. In particular, it has been shown that electric stimulation of the vagus nerve attenuates the inflammation during endotoxemia in rats [3], and that acetylcholine (ACh), the main parasympathetic neurotransmitter, effectively deactivates peripheral macrophages and inhibits the release of pro-inflammatory mediators, including the cytokine tumor necrosis factor-α (TNF-α). The ACh-dependent macrophage deactivation is mediated by the α7 subunit of the nicotinic ACh receptor (herein referred as α7 subunit), which is expressed in peripheral macrophages and has been described as essential for the so called "cholinergic anti-inflammatory pathway" [4,5].

Neuronal acetylcholine receptors (nAChRs) are ligand-gated ion channels, which belong to a large family of neurotransmitter receptors that includes the GABA_A_, glycine and 5-HT_3 _receptors [6]. Each nAChR consists of five homologous or identical subunits arranged around a central ion channel whose opening is controlled by ACh, nicotine and other receptor agonists [6]. At least 8 α subunits (α2–9) and three β subunits (β2–4) have been identified and the combinatorial association of different α and β subunits results in a variety of nAChRs [7].

In addition to neurons and peripheral macrophages, several studies have demonstrated the expression of nAChRs in cell types both within and outside the nervous system [8]. In the CNS, the presence of nAChRs has been demonstrated in O_2_A-oligodendrocyte precursor cells but not in adult differentiated oligodendrocytes, suggesting that receptor expression is developmentally regulated [9]. Cultured hippocampal astrocytes express functional α7 receptors [10] and cortical astrocytes express both nicotinic and muscarinic receptors [11]. A functional α7 nicotinic receptor has been recently described in murine microglial cells [12]. In peripheral organs, human and rat epithelial and endothelial cells express functional α7 receptors, as well as other nicotinic subunits such as α3, α5, β2 and β4 [13,14]. Acute or chronic exposure to nicotine has been shown to influence cell viability and motility of bronchial epithelial and endothelial cells [13]. Furthermore, nicotine has been shown to suppress the antimicrobial activities of murine alveolar macrophages [15]. Lymphocytes present both muscarinic and nicotinic receptors and it has been demonstrated that the interaction with antigen presenting cells enhances the synthesis and release of ACh [16]. These observations suggest that ACh might function as an important modulator of cellular interactions and immune functions.

Epidemiological studies indicate that nicotine, besides its immunosuppressive effects, may be protective against the development of neurodegenerative diseases such as Alzheimer disease (AD) and Parkinson's disease (PD) [17], in which a local inflammatory response is sustained by microglial cells, the largest population of phagocytes associated with the CNS. In normal healthy brain, microglial cells show a typical down-regulated or "resting" phenotype when compared to other tissue macrophages, but they rapidly react in response to a number of acute and chronic insults. Activated microglial cells could cause neuronal damage via liberation of free radicals as well as cytokines and toxic factors. Alternatively, microglia can exert neuroprotective functions by secreting growth factors or diffusible anti-inflammatory mediators, which contribute to resolve inflammation and restore tissue homeostasis [18,19]. Thus, understanding the molecular mechanisms governing microglial activation is essential to prevent tissue damage related to excessive activation. Since nicotine and ACh have been recently reported to inhibit TNF-α production in mouse microglial cultures, the aim of our study was to extend our knowledge on the effect of α7 subunit stimulation on the functional state of activated microglia. We first confirmed that rat microglia express the α7 subunit and we demonstrated that, in addition to inhibit TNF-α, the α7 agonist nicotine significantly up-regulated COX-2 expression and PGE_2 _synthesis. Other important microglial products, such as interleukin-1β (IL-1β), nitric oxide (NO) and interleukin-10 (IL-10) were not affected or moderately decreased.

## Materials and methods

### Reagents

All cell culture reagents were from Gibco (Grand Island, NY, U.S.A) and virtually endotoxin free (less then 10 E.U./ml as determined by the manufacturer). BCA protein assay was from Pierce (Rockford, Illinois). ELISA-kits for rat TNF-α and IL-10 were from Endogen Inc. (Woburn, MA). ED-1 monoclonal antibody was from Serotec (Oxford, UK). (±) Nicotine, α-bungarotoxin, FITC-α-bungarotoxin and lipopolysaccharide LPS (from Escherichia coli, serotype 026:B6) were from Sigma Chemical (St.Louis, MO). Rabbit polyclonal antibody against alpha 7 subunit was from Santa Cruz Biotechnology.

### Cell cultures

Microglial cultures were prepared from 10–14 day mixed primary glial cultures obtained from the cerebral cortex of 1-day-old rats, as previously described [20] and in accordance with the European Communities Council Directive N. 86/609/EEC. Microglial cells, harvested from the mixed primary glial cultures by mild shaking, were resuspended in Basal Eagle's Medium (BME) supplemented with 10 % fetal calf serum, 2 mM glutamine and 100 μg/ml gentamicin, and plated on uncoated plastic wells at a density of 1.25 × 10^5 ^cells/cm^2^. Cells were allowed to adhere for 20 min and then washed to remove non-adhering cells. After a 24 h of incubation, the medium was replaced with fresh medium containing the substance(s) under study. Cell viability was greater than 95%, as tested by Trypan Blue exclusion. Immunostaining, performed as previously described [20], revealed that cultures consisted of ≥ 99% positive cells for the microglia/macrophage marker ED1. Microglial cells were pre-stimulated for 30 min with nicotine and then stimulated for 24 h in the presence of 10 ng/ml LPS. A rat pheochromocytoma cell line, PC12, was propagated and maintained in RPMI-1640 medium supplemented with 5% heat-inactivated fetal bovine serum (FBS) and 10% heat-inactivated horse serum (HS) 100 U/ml penicillin, 100 μg/ml of streptomycin, and 2 mM L-glutamine. The cells were plated in 12-well plates for 24 h before performing RNA extraction.

### Cytokines nitric oxide and PGE_2 _determination

At the end of the incubation time, cell supernatants were collected, centrifuged, and stored at -70°C until tested. The levels of TNF-α and IL-10 were assayed by specific ELISAs, following the manufacturer's instructions. The ranges of determination were: 31–2500 pg/ml for TNF-α, 10–1000 pg/ml and 8–500 pg/ml for IL-10. The production of NO by measuring the content of nitrite, one of the end products of NO oxidation, as previously described [21]. PGE_2 _content was quantified using a specific radioimmunoassay [21]. The assay detection limit was 25 pg/ml and cross-reactivity of the antibody for PGE_2 _with other prostaglandins less than 0.25%.

### Immunostaining of microglial cells with α-bungarotoxin and western blot analysis

Microglial cells were plated on uncoated glass coverslips (2.5 × 10^5 ^cells/cm^2^), allowed to adhere for 20 min and then washed to remove non-adhering cells. After a 24 h of incubation, the complete BME medium was replaced with fresh BME medium without serum. Cells were incubated at 4°C for 15 min with FITC-labeled α-bungarotoxin at 1.5 μg/ml. Where indicated, nicotine was added at the concentration of 500 μM for 10 min, in order to saturate all the binding sites before the addition of FITC-labeled α-bungarotoxin. Cells were washed 3 times with BME medium and then fixed with 4% paraformaldehyde at room temperature for 15 min. After fixation, coverslips were washed twice with PBS solution, mounted in PBS:glycerol and examined using a fluorescent microscope. Cell culture lysates from microglial cells and PC12 cells (used as positive control) were analyzed for α7 subunit expression. Total protein content was estimated using the Bio-Rad protein assay. An aliquot corresponding to 50 μg (microglia cells) and 20 μg (PC12 cells) of total protein for each sample was separated by sodium dodecyl sulphate polyacrylamide gel elecrophoresis (SDS-PAGE) and transferred electrophoretically to nylon membranes. Membranes were blocked with 10% non-fat milk and incubated with a rabbit policlonal antibodies against α7 subunit (1:2000) overnight at 4°C. Horseradish peroxidase conjugated anti-rabbit IgG (1:5000, 1 h at 25°C) and ECL reagents were used as detection system.

### RNA extraction and semiquantitative RT-PCR analysis

Total RNA was prepared from rat microglia, PC12 cells and rat hippocampus using Trizol reagent according to manufacturer's protocol. Two μg of denatured total RNA were converted into first-strand cDNA using the SuperScript™synthesis system (Life Technologies™) in a total reaction volume of 20 μl following the conditions provided by the manufacturer's protocol.

Oligonucleotide primers with similar Tm were designed to generate a PCR fragment of 754 bp for the α7 subunit. PCR conditions (number of cycles and cDNA and primer concentration) that ensure the data to be obtained within the exponential phase of amplification of each template were carefully assessed. The amplification of the β-actin, COX-2 and α7 subunit within the exponential phase of amplification was achieved with 25, 30 and 40 cycles respectively.

Five μl, 15 μl and 40 μl of diluted cDNAs were amplified for β-actin, COX-2 and α7 respectively. PCR-amplification was done in a final volume of 50 μl containing 1x PCR buffer, the four dNTPs (0.2 mM), MgSO_4 _(2 mM), 1 Unit of Platinium Taq DNA polymerase High Fidelity (Invitrogen). The primers were: α7 subunit (Gene bank accession number **S53987**), sense 5'-TCT GTG CCC TTG ATA GCAC, antisense 5'-CTT CAT GCA ACC AGG ATC AG, product length 754; COX-2 [22], sense 5'-TGA TGA CTG CCC AAC TCC CATG; antisense 5'-AAT GTT GAA GGT GTC CGG CAGC, product length 702 bp; β-actin (accession number **NM031144**) sense 5'-GTC GAC AAC GGC TCC GGC ATG; antisense 5'-CTC TTG CTC TGG GCC TCG TCGC, product length 158 bp. A sample containing all reaction reagents except cDNA was used as PCR negative control in each experiment. The absence of genomic DNA was verified using 2 μg of RNA from microglia that was reverse-transcribed without the enzyme (-RT). The PCR conditions for COX-2 were as follows: initial denaturation at 94°C for 2 min followed by 30 cycles of 94°C for 30 sec, 58°C for 45 sec, 68°C for 1 min, and an additional cycle with extension at 72°C for 7 min. The PCR conditions for β-actin were as follows: initial denaturation at 94°C for 5 min followed by 25 cycles of 94°C for 30 sec, 68°C for 30 sec, 68°C for 45 sec and an additional cycle with extension at 72°C for 1 min. The PCR conditions for α7 subunit were as follows: initial denaturation at 94°C for 5 min followed by 40 cycles of 94°C for 30 sec, 57°C for 1 min, 68°C for 45 sec and an additional cycle with extension at 72°C for 7 min.

PCR products were analyzed by electrophoresis, stained with ethidium bromide and photographed. Transcript levels were analyzed by Fluor-STM Multimager analyser (Biorad). For each experiment, the ratio between optical density (arbitrary units) of bands corresponding to COX-2 and β-actin (used as internal standard) was calculated to quantify the level of the transcripts for COX-2 mRNAs.

### Statistical analysis

Data are expressed as mean ± SEM with the number of independent experiments, run in duplicate, indicated in figure legends. Comparison between treatment groups was made by Student's *t*-test. A two-tailed probability of less than 5 % (i.e. p < 0.05) was taken as statistically significant.

## Results

### Expression of α7 subunit mRNA in microglial cultures

**Figure 1:**
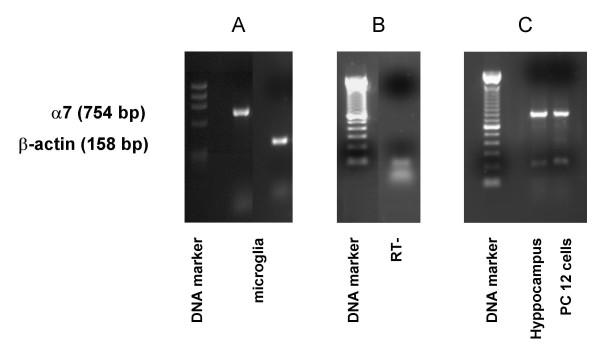
α7 nAChR subunit is expressed in rat microglial cultures. Semiquantitative RT-PCR analysis of α7 nAChR mRNA expression in rat microglial cells (A) and in PC12 cells and rat hippocampus (C). A 754-bp band corresponding to α7 nAChR was specifically amplified (acc. number **S53987**; amplified region: 906–1660). Expression of β-actin is shown as internal control. No contamination of genomic DNA was present as shown in panel B (-RT: RNA from microglia that was reverse transcribed without the enzyme and amplified for α7 subunit).

**Figure 2:**
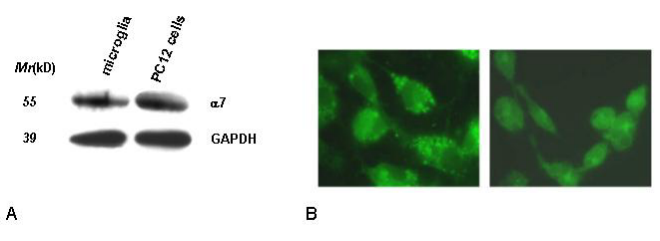
Western blot and fluorescent immunostaining of α7 nAChR in rat microglial cultures. A: Proteins from microglial cultures and PC12 cells were analysed by western blot (50 ug/lane) using specific polyclonal anti AChRα7 antibodies. B: microglial cells were pre-incubated in the absence (B, left panel) or presence of 500 μM nicotine (B, right panel) for 10 min and then incubated with FITC-labeleled-α-Bgtx (1.5 μg/ml) for 15 min at 4°C. A strong binding of α-Bgtx was observed on the cell surface of microglial cells. Nicotine pre-treatment resulted in a marked reduction of the intensity of binding.

The expression of the mRNA for α7 subunit in rat microglial cells was investigated by RT-PCR. As shown in Figure 1A, we detected a band of the expected size of 754-bp, which was then confirmed to correspond to α7 subunit by sequencing (M-Medical, Pomezia, I). The absence of genomic DNA contamination was demonstrated amplifying 2 μg of total RNA from microglia that was reverse-transcribed without the enzyme (Fig. 1B). As positive controls, we analyzed the expression of α7 subunit mRNA in rat hippocampus and PC12 cells (Fig. 1C), known to express the α7 subunit at high levels [23,24]. The expression of α7 subunit at protein level was established by western blot analysis using a specific antibody for the α7 subunit, which recognized a clear band with a molecular mass of approximately 55 kD from both microglial cells and PC12 cells, used as a positive control (Fig. 2A). The expression of the receptor was confirmed by labeling microglial cells with FITC-labeleled-α-bungarotoxin (α-Bgtx), a selective nicotinic antagonist. Microglial cells were pre-treated for 10 min in the absence (Fig. 2B, left panel) or in the presence (Fig 2B, right panel) of nicotine (500 μM) before adding 1.5 μg/ml FITC-α-Bgtx. As shown in Figure 2, a strong binding of α-Bgtx was observed on the cell surface of microglial cells (left panel), while nicotine pre-treatment resulted in a marked reduction of the intensity of the fluorescent signal (right panel).

### Effects of nicotine and α7 subunit activation on TNF-α release by rat microglial cells

Once we had demonstrated the presence of α7 subunit mRNA and protein in microglial cells, we studied the functional consequences of receptor activation using the specific agonist nicotine. Microglial cells were pre-treated for 30 min with increasing concentrations of nicotine and then incubated for 4 or 24 h in the absence or the presence of 10 ng/ml LPS. In resting microglial cultures nicotine did not affect the basal level TNF-α(data not shown).

**Figure 3:**
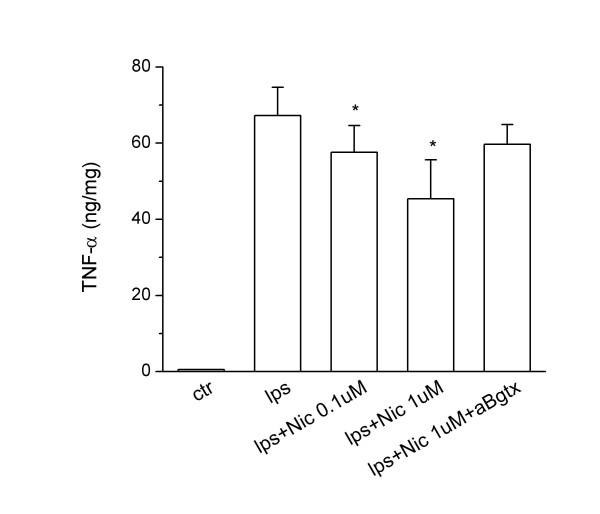
Effects of specific α7 nAChR agonist and antagonist on TNF-α production by activated rat microglial cultures. Microglial cells were subcultured for 24 h in 10% FCS-containing medium, which was replaced with fresh medium before stimulation. Nicotine (0.1–1 μM) and/or α-Bgtx were added 30 min before LPS stimulation (10 ng/ml). Supernatants were collected after 4 h and analyzed for TNF-α content. Data are shown as mean ± SEM for 3 independent experiments, run in duplicate. *p < 0.03 vs LPS.

As previously demonstrated using mouse microglial cultures, nicotine pre-treatments dose-dependently inhibited the release of TNF-α(Fig. 3). At 1 μM concentration, nicotine reduced the release of TNF-α after 4 h of LPS stimulation by approximately 35%, an effect similar to that recently reported for murine microglial cultures [12]. The inhibitory effect of nicotine on TNF-α release was still significant in microglial cultures exposed to LPS for 24 h (data not shown).

To verify that the effect of nicotine was mediated by α7 subunit, we measured the level of TNF-α in activated microglial cells exposed to nicotine in the presence or in the absence of α-Bgtx. The addition of 0.01 μM α-Bgtx almost totally prevented the inhibitory effect of nicotine (Fig. 3).

In addition to TNF-α, we also analyzed the release of two important microglial mediators such as NO and IL-1β and we found that nicotine pre-treatment only slightly reduced the release of NO (9 ± 4 and 14 ± 6 % of inhibition vs LPS activated microglia; n = 9; p < 0.04, for 1 and 10 μM nicotine, respectively) and did not modify the release of IL-1β (data not shown).

### Effects of nicotine and α7 subunit activation on interleukin-10 and prostaglandin E_2 _synthesis by rat microglial cells

We then analyzed the effects of nicotine on the production of interleukin-10 (IL-10) and prostaglandin E_2 _(PGE_2_), two important local mediators with anti-inflammatory and immunoregulatory functions. Nicotine pre-treatment only moderately reduced (18.6 ± 7% of inhibition vs LPS activated microglia; n = 4; p < 0.03, for 1 μM) the level of IL-10 in the culture media of microglia cells stimulated for 24 h with LPS (data not shown).

**Figure 4:**
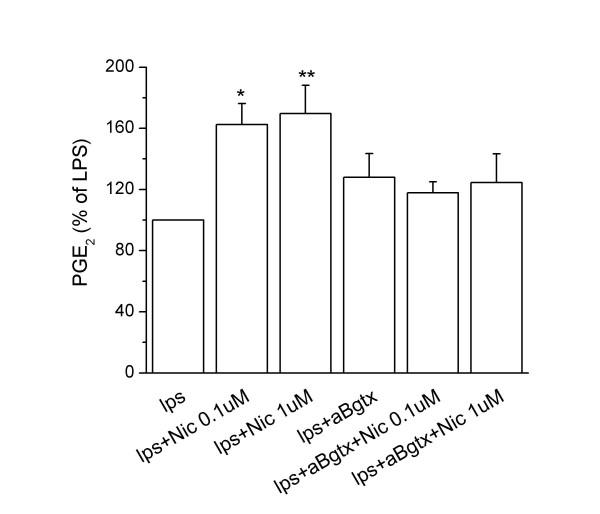
Effect of specific α7 nAChR agonist and antagonist on PGE_2 _synthesis by activated rat microglial cultures. Microglial cells were subcultured as in Fig. 3, and nicotine (0.1–1 μM) added 30 min before LPS stimulation (10 ng/ml). Supernatants were collected after 24 h and analyzed for PGE_2 _content. Data, with induction expressed as a percentage of LPS-induced PGE_2 _production, are shown as mean ± SEM for 5 independent experiments, run in duplicate. The levels of PGE_2 _were undetectable in basal conditions, and were 24 ± 6 ng/mg protein after LPS-stimulation for 24 h. *p < 0.05 vs LPS; **p < 0.02 vs LPS.

**Figure 5:**
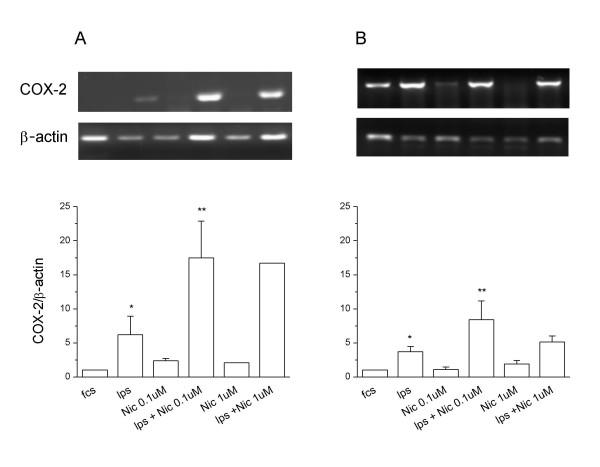
Semiquantitative RT-PCR analysis of COX-2 mRNA. Representative semi-quantitative RT-PCR analysis of COX-2 mRNA in microglial cultures, subcultured as in Fig. 3, pre-treated with nicotine (Nic, 0.1–1 μM) for 30 min and stimulated for 7 h (A, upper panels) or 24 h (B upper panels) with LPS (10 ng/ml). The amount of COX-2 mRNA, expressed as the ratio of densitometric measurement of the sample to the corresponding internal standard (β-actin), is shown in the lower panels. Data are shown as mean ± SEM for 3 to 4 independent experiments, with the exception of 1 μM nicotine, panel A (n = 2); all run in duplicate. * p < 0.05 vs fcs; **p < 0.05 vs fcs.

By contrast, nicotine pre-treatments dose-dependently enhanced the synthesis of PGE_2 _in LPS-activated microglial cells. The presence of 0.01 μM α-Bgtx, blocked the nicotine-dependent increase of PGE_2 _released by LPS-activated microglia (Fig. 4). At this concentration, α-Bgtx did not by itself affect basal (not shown) or LPS-induced PGE_2_. We investigated the molecular mechanism underlying the increased synthesis of PGE_2 _induced by α7 subunit stimulation by measuring by RT-PCR the levels of COX-2 mRNA. COX-2 is the enzyme responsible for the first committed step in prostaglandin synthesis, and is known to be readily induced by LPS in both peripheral macrophages and microglia [25]. As expected, COX-2 mRNA was expressed at low levels in resting microglial cultures and was remarkably increased after 7 h and 24 h of LPS treatment (Fig. 5). The basal COX-2 mRNA level was not significantly altered by nicotine pre-treatment at any tested concentration (0.1 μM and 1 μM) or incubation time (7 and 24 h). However, nicotine pre-treatment strongly increased the levels of COX-2 mRNA induced by 7 h treatment with 10 ng/ml LPS; the maximal effect was reached at 0.1 μM concentration (Fig. 5A). The enhancing effect of nicotine pre-treatment persisted after 24 h of LPS-treatment, although the increase was significant only at the lower concentration of nicotine (Fig. 5B).

## Discussion

The present study provides evidence that supports the existence of a cholinergic control of microglial activation. First, we have confirmed using rat microglial cells previous data showing that murine microglia express the α7 subunit and that their exposure to the specific agonist nicotine reduces LPS-induced release of the pro-inflammatory molecule TNF-α, thus suggesting that these events are not species specific.

Furthermore, we extended the analysis of α7 subunit activation to other important microglial functions, including the synthesis of mediators possessing anti-inflammatory and immunomodulatory activities. We found that in LPS-activated microglial cells, the interaction of α7 subunit with its agonist nicotine had moderate or no effect on the release of NO, IL-1β and IL-10. By contrast, nicotine treatment significantly increased the expression of COX-2 and the synthesis of PGE_2_. The effect of nicotine on the LPS-induced PGE_2 _release was significantly reversed by the specific antagonist of α7 subunit, α-bungarotoxin, demonstrating the involvement of α7 nicotinic receptors in the induction of PGE_2 _production by activated microglial cells.

COX-2 is the inducible isoform of the enzyme responsible for the first committed step in PGE_2 _synthesis, one of the major prostaglandins produced during inflammatory response and potent modulator of several macrophage and lymphocyte functions [26]. Within the brain, COX-2 activity and PGE_2 _production, depending on their levels of induction, have been associated with both protective and harmful effects on neurons and glial cells [27]. In microglial cells, COX-2 is the major isoform, rapidly induced by LPS stimulation or interaction with apoptotic neurons [28]. The constitutive isoform COX-1 is only moderately expressed by these cells and is not up-regulated during their activation [25,27].

PGE_2 _has been found to be neuroprotective in several experimental settings. At nanomolar concentrations, PGE_2 _protects hippocampal and cortical neuronal cultures against excitotoxic injury or LPS-induced cytotoxicity [29-32]. In hippocampal neuronal and organotypic cultures, the protective effect of PGE_2 _against glutamate and oxygen deprivation is mediated by the activation of the EP2 receptor, one of the four PGE_2 _receptor subtypes whose activation leads to cAMP formation [31]. The protective effect of EP2 receptor activity has been confirmed in vivo, in a model of transient forebrain ischemia, in which the genetic deletion of this PGE_2 _receptor exacerbates the extent of neuronal damage [31]. On the other hand, at concentrations in the μM – mM range, PGE_2 _contributes to neuronal death and stimulates release of glutamate by astrocytes [33-35].

PGE_2 _has also been shown to down-regulate microglial activation and expression of pro-inflammatory genes, including TNF-α, both in vitro and in vivo [36,37]. We have recently found that the interaction of microglial cells with apoptotic neurons promotes the synthesis of PGE_2 _along with neuroprotective and immunoregulatory molecules such as TGF-β and NGF [38,28]. In this system, the release of PGE_2 _is triggered by the specific interaction between phosphatidylserine, a phospholipid exposed on the cell surface during the initial phase of apoptosis, with its cognate receptor expressed by microglia [39], consistent with previous studies on peripheral macrophages [40]. It has been suggested that the PGE_2_, released by macrophages engulfing apoptotic cells, contributes to one of the main features of apoptotic cell death, namely the efficient removal of dying cells without eliciting inflammation in the surrounding tissue [41]. It is therefore tempting to speculate that the α7 subunit-dependent increase of PGE_2 _in activated microglia cells is part of an anti-inflammatory pathway regulated by the cholinergic system. The detection of microglial cells, astrocyte processes and choline acetyltransferase- (ChAT-) positive fibers around β-amyloid plaques in transgenic APP_SW _mice suggests a close connection between cholinergic terminals and microglial cells [42]. A deficit in ACh level due to loss of cholinergic neurons associated with AD as well as aging could contribute to the establishment of chronic inflammation rendering microglial cells more susceptible towards environmental changes and orientating them towards a pro-inflammatory phenotype. However, to date there is no definitive evidence of a causal link between loss of cholinergic neurons and increased levels of pro-inflammatory cytokines such as TNF.

In the last few years, several lines of evidence have suggested that activation of α7 subunits plays an important role in the maintenance of cognitive functions in several neurodegenerative disorders [43]. Epidemiological studies have shown that cigarette smoking can be protective against the development of AD, PD and other types of dementia, suggesting that chronic inhalation of nicotine may slow the progression of these neurodegenerative diseases or improve some cognitive responses in AD patients [44,17]. Loss of nAChRs has been reported in patients with diverse forms of dementia [45]. In particular, a reduction in α7 subunit number was detected in AD and PD brain tissue specimens [46]. The administration of ligands targeting nicotinic receptors in animal models of neurodegeneration, as well as in humans, induced cognitive improvement [47] and conferred neuroprotection against several neurotoxic agents [48,49]. Furthermore, cholinesterase inhibitors used in the symptomatic treatment of AD have been reported to exert additional benefits through the increased density of specific nicotinic receptor subunits (including the α7) [50]. This effect could be relevant in view of the anti-inflammatory role suggested for the α7 subunit.

As mentioned in the introduction, the presence of α7 subunit on immune cells as well as on other non-excitable cells has provided a molecular basis for a non-neuronal cholinergic pathway that might function as an essential regulator of inflammation as well as immune responses [4,5]. Primary cultures of astrocytes and microglia show ChAT activity and synthesize acetylcholine [51]. Accordingly, we have found the expression of ChAT mRNA in both resting and activated microglia cells (unpublished results). This suggests that this neurotransmitter may act as a local hormone and contribute to the regulation of microglial functions.

It should be noted that although our study focused on the effects of nicotine on the process of microglial activation induced by LPS, our findings may have broader implications since other microglial activators, such as pro-inflammatory cytokines and fibrillogenic peptides, share some common signaling pathways with LPS [52,53]. In addition, it has been recently reported that the LPS receptor CD14 interacts with fibrils of Alzheimer amyloid peptide and a deficiency of this receptor significantly reduces fibril-induced microglial activation [54].

At present, the signaling pathways downstream to α7 subunit activation and leading, in particular, to COX-2 and PGE_2 _up-regulation is under investigation. Shytle *et al. *[12] have reported that either ACh or nicotine inhibit LPS-induced phosphorylation of the mitogen-activated protein kinases p44/42 and p38 in murine microglia. We have recently found a reduction of p38 phosphorylation in two experimental settings in which exposure of microglial cells to phosphatidylserine vesicles – mimicking apopototic neurons – or to chronic activation stimuli, resulted in downregulation of pro-inflammatory cytokines and in enhancement of PGE_2 _synthetic pathway [55,56], thus suggesting that p38 may also have a role in α7 dependent up-regulation of COX-2.

## Conclusions

Activation of α7 nicotinic receptors in microglial cells by nicotine controls some important microglial functions, thus preventing chronic inflammation. Since microglial activation and chronic inflammation have been associated with most neurodegenerative pathologies [57] the understanding of the molecular pathway(s) triggered by α7 subunit activation in microglial cells will offer new venues for potential pharmacological regulation of microglial activation in neurodegenerative diseases. At the same time, the development of molecules able to stimulate the α7 subunit may represent a potential promising approach for the treatment of these disorders.

## List of abbreviations

Lipopolysaccharide (LPS)

Acetylcholine (ACh)

Neuronal acetylcholine receptors (nAChRs)

Tumor necrosis factor-α (TNF-α)

Prostaglandin E_2 _(PGE_2_)

Interleukin-1β (IL-1β)

Nitric oxide (NO)

Interleukin-10 (IL-10)

## Competing interests

The author(s) declare that they have no competing interests.

## Authors' contributions

RDS conceived of the study, participated in its design and coordination, produced the primary microglial cultures, performed the ELISA and the immunofluorescence staining, was primarily responsible of the RT-PCR review the data and drafted the manuscript. MAAC participated in the design and coordination of the study, produced the primary microglial cultures, performed the ELISA and the immunofluorescence staining, was primarily responsible for western blot analysis and review the data. DC participated in the production of the primary microglial cultures and in RT-PCR. LM contributed to the design of the study, guided data interpretation and presentation and assisted in the preparation of the manuscript.
